# Evidence of diversity and recombination in *Arsenophonus* symbionts of the *Bemisia tabaci* species complex

**DOI:** 10.1186/1471-2180-12-S1-S10

**Published:** 2012-01-18

**Authors:** Laurence Mouton, Magali Thierry, Hélène Henri, Rémy Baudin, Olivier Gnankine, Bernard Reynaud, Einat Zchori-Fein, Nathalie Becker, Frédéric Fleury, Hélène Delatte

**Affiliations:** 1 Université Claude Bernard Lyon 1, Laboratoire de Biométrie et Biologie Evolutive, UMR CNRS 5558, 43 Bd du 11 Novembre 1918, 69622 Villeurbanne Cedex, France; 2 CIRAD, UMR Peuplements Végétaux et Bioagresseurs en Milieu Tropical, 3P, 7 chemin de l'IRAT 97410 Saint Pierre La Réunion, France; 3 Université de Ouagadougou, Unité de Formation et de Recherche en Sciences de la Vie et de la Terre (UFR-SVT), Laboratoire d’Entomologie Fondamentale et Appliquée, 09 848 Ouagadougou 09, Burkina Faso; 4 Agricultural Research Organization, Department of Entomology, Newe Ya'ar Research Center, PO Box 1021, Ramat Yishay 30095, Israel; 5 Museum National d’Histoire Naturelle, UMR CNRS 7205 Origine, Structure et Evolution de la Biodiversité, CP 50, 57 rue Cuvier, 75231 Paris Cedex 05, France

## Abstract

**Background:**

Maternally inherited bacterial symbionts infecting arthropods have major implications on host ecology and evolution. Among them, the genus *Arsenophonus* is particularly characterized by a large host spectrum and a wide range of symbiotic relationships (from mutualism to parasitism), making it a good model to study the evolution of host-symbiont associations. However, few data are available on the diversity and distribution of *Arsenophonus* within host lineages. Here, we propose a survey on *Arsenophonus* diversity in whitefly species (Hemiptera), in particular the *Bemisia tabaci* species complex. This polyphagous insect pest is composed of genetic groups that differ in many ecological aspects. They harbor specific bacterial communities, among them several lineages of *Arsenophonus*, enabling a study of the evolutionary history of these bacteria at a fine host taxonomic level, in association to host geographical range and ecology.

**Results:**

Among 152 individuals, our analysis identified 19 allelic profiles and 6 phylogenetic groups, demonstrating this bacterium's high diversity. These groups, based on *Arsenophonus* phylogeny, correlated with *B. tabaci* genetic groups with two exceptions reflecting horizontal transfers*.* None of three genes analyzed provided evidence of intragenic recombination, but intergenic recombination events were detected. A mutation inducing a STOP codon on one gene in a strain infecting one *B. tabaci* genetic group was also found. Phylogenetic analyses of the three concatenated loci revealed the existence of two clades of *Arsenophonus.* One, composed of strains found in other Hemiptera, could be the ancestral clade in whiteflies. The other, which regroups strains found in Hymenoptera and Diptera, may have been acquired more recently by whiteflies through lateral transfers.

**Conclusions:**

This analysis of the genus *Arsenophonus* revealed a diversity within the *B. tabaci* species complex which resembles that reported on the larger scale of insect taxonomy. We also provide evidence for recombination events within the *Arsenophonus* genome and horizontal transmission of strains among insect taxa. This work provides further insight into the evolution of the *Arsenophonus* genome, the infection dynamics of this bacterium and its influence on its insect host's ecology.

## Background

Many arthropods live in symbiosis with one or more endosymbiotic bacteria, establishing a wide diversity of symbiotic associations ranging from mutualism to parasitism [1,2]. When arthropod hosts feed on imbalanced diets, such as plant sap or vertebrate blood, mutualistic bacterial symbionts play a central role in their biology by providing essential nutrients that are lacking or limited [3], leading to obligatory cooperative insect-microbial relationships.

Arthropods also harbor facultative symbionts acquired more recently, leading to complex associations with shorter epidemiological and evolutionary dynamics [4,5]. These are mainly vertically transmitted but according to the host-symbiont association, horizontal transfers may occur within and between species on different evolutionary time scales [6-9]. An extremely diverse group of bacterial taxa is involved in facultative symbiosis, with a wide range of both hosts and phenotypes. Some facultative endosymbiotic bacteria confer direct fitness benefits such as protection against natural enemies [10,11], host-plant specialization [12] or thermal tolerance [13]. Others, like the alphaproteobacterium *Wolbachia* and the Bacteroidetes *Cardinium*, manipulate host reproduction to enable their spread and maintenance in host populations despite deleterious effects (for review see Stouthamer *et al. *[14]).

Among the symbiotic bacteria, the gammaproteobacterium genus *Arsenophonus* has particular characteristic features with regard to lineage diversity, host spectrum and the symbiotic relationships established with its host. It thus constitutes a good model to study the evolutionary process shaping symbiotic associations. The diversity of *Arsenophonus* host species is particularly large, including insects, other arthropods (such as ticks) and plants [15]. This can be explained by the symbiont's transmission routes since this vertically transmitted bacterium can also be acquired by horizontal transfer within and among species [16,17]. Moreover, some strains can be cultivated on cell-free cultures [18]. *Arsenophonus*-host relationships range from parasitism to mutualism, with the induction of various phenotypes such as reproductive manipulation (male-killing) [19], phytopathogenicity [20] or obligatory mutualism [21,22]. However, in most reported symbiotic associations, the impact of this symbiont on the host phenotype remains unknown. Based on *rRNA* gene analysis, phylogenetic studies have revealed an extremely high diversity of bacterial lineages forming a monophyletic group [15]. In addition, the *Arsenophonus* phylogeny encompasses several other host-specific sub-clusters with lower divergence associated to ticks, plants, triatomine bugs, whiteflies, several genera of hippoboscids and ants, but no co-speciation pattern within clades. Beside these bacterial lineages that cluster according to host taxonomy, a number of closely related *Arsenophonus* strains infect unrelated host species. Moreover, the same host species sometimes harbors several *Arsenophonus* lineages, a pattern that is probably due to the *Arsenophonus*'s ability to be horizontally transferred, as recently demonstrated in the hymenopteran parasitoids of the family Pteromalidae [17]. Previous studies have shown that whitefly species can host different strains of several bacteria [15,23,24] , and they thus appear to be particularly relevant to investigating *Arsenophonus* diversity and evolution. However, we cannot disregard the fact that *rRNA*-based phylogeny suffers inconsistencies as a result of intragenomic heterogeneity among the 8 to 10 estimated *rRNA* copies in the *Arsenophonus* genome [25]. Moreover, biased phylogeny can also result from homologous recombination, which appears more frequently in symbiotic bacteria than expected based on their intracellular lifestyle and vertical transmission [26,27]. The availability of the complete sequence of the *Arsenophonus* genome now provides the opportunity to perform a more accurate exploration of the evolutionary history and ecological spread of this pervasive symbiotic bacterium on different host-taxonomical scales.

Among the whiteflies, the *Bemisia tabaci* (Homoptera, Aleyrodidae) species complex has emerged as a focus of attention for several reasons, chief among them being the ongoing species radiation and the high prevalence of a wide diversity of endosymbiotic bacteria, including several lineages of *Arsenophonus *[28]. The whitefly *B. tabaci* is a worldwide polyphagous pest of vegetables and ornamental crops, previously thought to be a unique species composed of several well-differentiated genetic groups or biotypes. Recently however, some of these groups have been recognized as true species, so that *B. tabaci* is now considered a complex of 24 cryptic species which barely interbreed and form different phylogenetic clades [29]. The biological data needed to draw clear boundaries among species and to identify the cause of such genetic differentiation are still lacking. This phloem-feeding insect harbors a primary symbiont, *Portiera aleyrodidarum*, required for supplementing its specialized diet. *B. tabaci* also hosts up to six vertically transmitted secondary symbionts, some of which are phylogenetically highly distant [23]. For each of these symbionts, the phenotypic consequences of infection in *B. tabaci* remain poorly identified, if at all [30]. Nevertheless, in other insect species, some of these bacteria are known to manipulate host reproduction, while others increase resistance to natural enemies [4,10,14,31]. Moreover, the symbionts are thought to play a major role in the viral transmission capacities of the pest [32,33]. Interestingly, multiple bacterial infections are common in *B. tabaci*, and the endosymbiotic community is correlated with the *B. tabaci* genetic groups on different scales of differentiation [28,34,35]. This raises the question of these endosymbionts role in *B. tabaci* biology and species radiation. Within the 24 well-differentiated *mtDNA* groups recognized as true species by De Barro *et al*. [29] and that regroup all previously described biotypes, *Arsenophonus* has been found in AsiaII3 (ZHJ1 biotype), AsiaII7 (Cv biotype), Indian Ocean (Ms biotype), Mediterranean [Q and Africa Silver Leafing (ASL) biotypes which probably form true species] and the Sub-Saharan Africa species [Africa non-Silver Leafing (AnSL) biotype] [28,34-38]. For all other species or groups, there is either no data or they have proven to be free from infection. For example, among the putative species of the Africa/Middle East/Asia Minor clade which contains the most invasive species the Ms, Q and ASL groups *Arsenophonus* appears well established, whereas the invasive B group has been shown to be uninfected, despite extensive symbiont screening [28,34,39]. The prevalence varies considerably within and among populations and genetic groups infected by *Arsenophonus*. For example, Q is composed of three *COI*-differentiated groups, Q1, Q2 and Q3 [28]. To date, these three cytotypes have not shown the same geographical distribution and show different endosymbiotic bacterial community compositions [28,40]. The subgroup Q1, found in Europe, is not infected by *Arsenophonus* but harbors three other bacteria [28]. In contrast, Q2 observed in the Middle East and Q3 reported only in Africa show high prevalence of *Arsenophonus* in co-infection with *Rickettsia *[28,34,41]. Ms individuals are highly infected by *Arsenophonus* with a high level of co-infection by *Cardinium *[37]. All of these groups (B, Q, ASL, Ms and AnSL) show quite different geographical ranges. Ms has been detected on the islands in the southwestern part of the Indian Ocean, Tanzania and Uganda, living in sympatry with B [42]. ASL and AnSL have been reported only in Africa [28,35,43-46]. In contrast, the invasive B and Q groups are spread all over the world. Q has been found in Africa, America, Europe, Asia and the Middle East [28,34,47,48]. However, this situation is constantly in flux, because commercial trade is responsible for recurrent introduction/invasion processes of *B. tabaci* giving rise to new sympatric situations. Moreover, potential horizontal transfers of symbionts and interbreeding can generate new nucleo-cytoplasmic combinations and thus rapid evolution of symbiont diversity.

Patterns of *Arsenophonus* infection in *B. tabaci* within the high-level Africa/Middle East/Asia Minor groups make this clade a good candidate to study, on fine taxonomic and time scales, the spread of this bacterium, its ability to be horizontally transferred and finally, its evolutionary history, including genetic diversity generated by recombination events. In the present paper, we explore the prevalence and diversity of *Arsenophonus* strains in this clade using an MLST approach to avoid the disadvantages of the *rRNA* approach. In parallel we also studied, as an outgroup, the Sub-Saharan AnSL species (S biotype), considered the basal group of this species complex, and two other whitefly species found at the sampling sites, *Trialeurodes vaporariorum* and *Bemisia afer*.

## Methods

### Insect sampling

**Figure 1:**
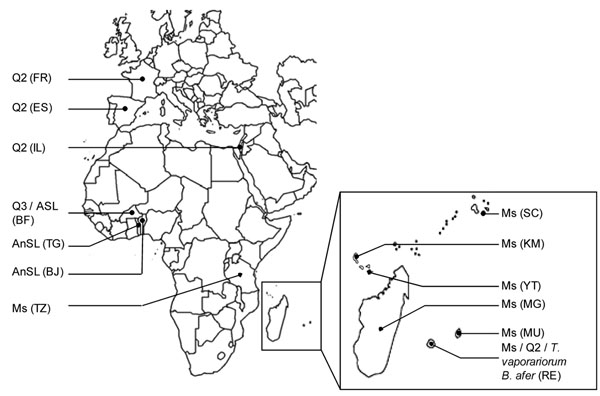
**Location of sampling sites indicating the presence of the genetic groups of *Bemisia tabaci* (Q2, Q3, AnSL, ASL, Ms), *Bemisia afer* and *Trialeurodes vaporariorum.*** Samples were collected in mainland France (FR), Spain (ES), Israel (IL), Burkina Faso (BF), Togo (TG), Benin (BJ), Tanzania (TZ), Seychelles (SC), Comoros Grande Comore (KM), Mayotte (YT), Madagascar (MG), Mauritius (MU) and Reunion (RE).

Individuals from different species of *Bemisia tabaci* and two other Aleyrodidae species were collected from 2001 to 2010 from various locations and host plants in Africa and Europe and stored in 96% ethanol (Table 1, Figure 1).

**Table 1:** Sampling locations of Aleyrodidae used in this study, ***B. tabaci*** genetic group or insect species, and ***Arsenophonus*** prevalence

Acronym	Country	Locality	Host plant	Year	Gen.gr. /species	ntot	*Arsen. *prev.	n	***fbaA***** Acc. no.**	***ftsK***** Acc. no.**	***yaeT***** Acc. no.**
O2	BF	Univ-Ouaga	*Lantana camara*	2008	Q3	33	100%	16	JF743134-49	JF743286-301	JF743438-53
B4	BF	Labo Minima	Tobacco	2007	Q3	20	80%	4	JF743071-74	JF743223-26	JF743375-78
										
					**Q3**	**53**		**20**			
B1	BF	Bobo/Kuinima	Tomato	2007	ASL	19	84%	10	JF743057-66	JF743209-18	JF743361-30
B2	BF	Bobo/Kuinima	Marrow	2007	ASL	11	82%	4	JF743067-70	JF743219-22	JF743371-74
										
					**ASL**	**30**		**14**			
Be8	BJ	Agonkanmey	Cassava	2007	AnSL	20	65%	3	JF743075-77	JF743227-29	JF743379-81
To2	TG	Zone portuaire	Cassava	2007	AnSL	20	35%	3	JF743203-05	JF743355-57	JF743507-09
										
					**AnSL**	**40**		**6**			
ISR	IL	Lab rearing		2010	Q2	6	100%	6	JF743119-24	JF743271-76	JF743423-48
FrOttA	FR	Frﾎjus	Hibiscus-Gr.	2010	Q2	6	50%	3	JF743112-14	JF743264-66	JF743416-18
SLVA	FR	St Laur. du Var	Hibiscus-Gr.	2010	Q2	8	63%	5	JF743186-90	JF743338-42	JF743490-94
VilCu	ES	Viladecans	Cucumber	2010	Q2	3	33%	1	JF743208	JF743360	JF743512
CaMe	ES	Cabrils	Melon	2010	Q2	14	72%	4	JF743084-87	JF743236-39	JF743388-91
BlaPe	ES	Blanes	Pepper	2010	Q2	20	80%	6	JF743078-83	JF743230-35	JF743382-87
SPAubF29	RE	St Pierre	Eggplant	2010	Q2	1	100%	1	JF743191	JF743343	JF743495
										
					**Q2**	**58**		**26**			
GC5	KM			2001	Ms	19	11%	1	JF7443115	JF743267	JF743419
DATO	MG	Diego	Tomato	2001	Ms	21	24%	3	JF743102-04	JF743254-56	JF743406-08
DCTO	MG	Diego	Tomato	2001	Ms	11	9%	1	JF743105	JF743257	JF743409
DITO	MG	Diego	Tomato	2001	Ms	27	22%	3	JF743107-09	JF743259-61	JF743411-13
DNTO	MG	Diego	Tomato	2001	Ms	7	29%	1	JF743110	JF743262	JF743414
DIAU	MG	Diego	Eggplant	2001	Ms	6	50%	1	JF743106	JF743258	JF743410
FDHC	MG	Ford Dauphin	Bean / Cucumber	2001	Ms	8	13%	1	JF743111	JF743263	JF743415
MI	MG	Miandrivazo	Eggplant	2001	Ms	10	10%	1	JF743127	JF743279	JF74343l
MOBE	MG	Morondave	Cabbage	2001	Ms	11	9%	1	JF743128	JF743280	JF743432
MOCO	MG	Morondave	Cucumber	2001	Ms	24	4%	1	JF743129	JF74328l	JF743433
TOTO	MG	Tamatave	Tomato	2001	Ms	18	28%	2	JF743206-07	JF743358-59	JF743510-11
TACH	MG	Tananarive	Cabbage	2001	Ms	8	38%	3	JF743193-95	JF743345-47	JF743497-99
TACO	MG	Tananarive	Zucchini	2001	Ms	8	25%	1	JF743196	JF743348	JF743500
TATO	MG	Tananarive	Tomato	2001	Ms	7	43%	1	JF743199	JF74335l	JF743503
TBAU	MG	Tulear	Eggplant	2001	Ms	7	43%	3	JF743200-02	JF743352-54	JF743504-06
MA10	MU	Solitude	Tomato	2001	Ms	4	100%	2	JF743125-26	JF743277-78	JF743429-30
MY6	YT	Tzoundzou	Tomato	2001	Ms	11	45%	1	JF743133	JF743285	JF743437
MY10	YT	Marembere	Tomato	2001	Ms	2	100%	1	JF743130	JF743282	JF743434
MY14	YT	Dzoumonie	West Indian *Lantana*	2001	Ms	5	56%	1	JF743131	JF743283	JF743435
MY17	YT	Kangani	Tomato	2001	Ms	3	75%	1	JF743132	JF743284	JF743436
SE6	SC	La Digue	Cassava	2001	Ms	8	35%	1	JF743164	JF743316	JF743468
SaEuph	RE	St Andre	Annual Poinsettia	2010	Ms	83	94%	8	JF743156-63	JF743308-15	JF743460-67
SaAub	RE	St Andre	Eggplant	2010	Ms	91	13%	4	JF743151-54	JF743303-06	JF743455-58
PiHar	RE	Petite Ile	Bean	2010	Ms	74	50%	1	JF743150	JF743302	JF743454
SGEuph	RE	St Gilles	Annual Poinsettia	2010	Ms	97	85%	21	JF743165-85	JF743317-37	JF743469-89
SREuph	RE	St Rose	Annual Poinsettia	2010	Ms	74	89%	1	JF743192	JF743344	JF743496
Tanzani 2.8	TZ	Morogoro	Tomato	2008	Ms	8	75%	1	JF743197	JF743349	JF743501
Tanzani 4.1	TZ	Arusha	Tomato	2008	Ms	8	75%	1	JF743198	JF743350	JF743502
										
					**Ms**	**660**		**68**			
haric	RE	Bras de Ponto	Bean	2010	*T. vaporar.*	10	100%	3	JF743116-18	JF743268-70	JF743420-22
Co_pl	RE	Tampon 14e	Zucchini field 1	2011	*T. vaporar.*	10	100%	7	JF743088-94	JF743240-46	JF743392-98
Co_p2	RE	Tampon 14e	Zucchini field 2	2011	*T. vaporar.*	10	100%	7	JF743095-101	JF743247-253	JF743399-405
										
					***T. vaporar.***	**30**		**17**			
SaAubF53	RE	St Andre	Eggplant	2010	*B. afer*	2	100%	1	JF743155	JF743307	JF743459
										
					***B. afer***	**2**		**1**			

								**152**			

*T. vaporar. : Trialeurodes vaporariorum. B. afer : Bemisia afer.* Country abbreviations stand for France (FR), Spain (ES), Israel (IL), Burkina Faso (BF), Togo (TG), Benin (BJ), Tanzania (TZ), Seychelles (SC), Comoros Grande Comore (KM), Mayotte (YT), Madagascar (MG), Mauritius (MU) and Reunion (RE). Gr.: greenhouse. Gen. gr. : Genetic group. ntot: number of individuals screened for *Arsenophonus*, n: number of individuals used for the phylogenetic analysis. *Arsen.* Prev.: *Arsenophonus* prevalence. Accession numbers are given for *fbaA*, *ftsK* and *yaeT* sequences obtained in this study.

### DNA extraction and PCR amplification

#### *Arsenophonus* detection and identification of *B. tabaci* genetic groups

Insects were sexed and DNA was extracted as previously described by Delatte *et al*. [49]. All samples were screened for *Arsenophonus* infection using the specific primers Ars-23S1/Ars-23S2 targeting the *23SRNA* gene [50] (Table 2). To check for extracted DNA quality, all samples were also tested for the presence of the primary symbiont *P. aleyrodidarum* using specific primers for the *16S rRNA* genes described by Zchori-Fein and Brown [23]. When positive signals were recorded in both PCRs, insects were used in the analysis. *B. tabaci* genetic groups were identified by PCR-RFLP (random fragment length polymorphism) test based on the mitochondrial marker *COI* (*Cytochrome Oxidase 1*) gene as described by Gnankine *et al*. [35] for Q, ASL and AnSL individuals. A set of 10 microsatellite markers was used to identify Ms according to Delatte *et al. *[42]. Moreover, a portion of the *COI* gene was sequenced for five individuals from each of the different *B. tabaci* genetic groups, using the protocol described by Thierry *et al*. [37] and Gnankine *et al*. [35] (Figure S1 in Additional file 1).

**Table 2:** Nucleotide sequences of primers used in this study.

rRNA Gene	Primers	Sequences	Tm	References
*23S*	Ars-23S1	5’- CGTTTGATGAATTCATAGTCAAA -3’	58°C	Thao & Baumann [50]
	Ars-23S2	5’- GGTCCTCCAGTTAGTGTTACCCAAC -3’		

*ftsK*	*ftsK*For1	5’- GCCGATCTCATGATGACCG -3’	59°C	This study
	*ftsK*Rev1	5’- CCATTACCACTCTCACCCTC -3’		
	*ftsK*For2	5’- GCTGATCTGATGATGACTG -3’		
	*ftsK*Rev2	5’- CCATTACTACCTTCACCATC -3’		

*yaeT*	*Yae*TF496	5’- GGCGATGAAAAAGTTGCTCATAGC -3’	55°C	This study
	*Yae*TR496	5’- TTTTAAGTCAGCACGATTACGCGG -3’		

*fbaA*	*fba*Af	5’- GCYGCYAAAGTTCRTTCTCC -3’	58°C	Duron *et al*. [17]
	*fba*Ar	5’- CCWGAACCDCCRTGGAAAACAAAA -3’		
	*fba*ARLM	5’- TTHARATTATTTTCCGCTGG -3’		This study

*COI*	COI-F-C1	5’- CATCTAATCAGCAGTGAGGCTGG -3’	57°C	Thierry *et al*. [37]
	COI-R-C1	5’- AAAAGTTAAATTTACTCCAAT -3’		

#### Study of *Arsenophonus* diversity

PCRs targeting three different genes of *Arsenophonus* were carried out on positive samples with two sets of primers designed specifically for this study (*ftsK*: *ftsk*For1/Rev1, *ftsk*For2/Rev2; *yaeT*: *Yae*TF496/*Yae*TR496, see Table 2) and one set from the literature (*fbaA*: *Fba*Af/*Fba*Ar) [17]. For the Q group, amplifications failed for some individuals and the primer *Fba*ArLM (Table 2) was then used instead of *Fba*Ar. These two primers are adjacent and their use permits the amplification of similar sequences. PCRs were performed in a final volume of 25 µL, with 10 ng of total DNA extract, 200 μM dNTPs, 200 nM (for *fbaA* and *yaeT*) or 300 nM (for *ftsK*) of each primer and one unit of proofreading DAp GoldStar (Eurogentec) or 0.5 unit of DreamTaq® DNA polymerase (Eurobio). For the DAp Goldstar *Taq* polymerase, MgCl_2_ was added at the following optimal concentrations: 1 mM for *fbaA* primers, 1.5 mM for *yaeT* primers and 2 mM for *ftsK* primers. All PCR amplifications were performed under the following conditions: initial denaturation at 95°C for 2 min followed by 35 cycles at 94°C for 30 s, 55°C to 59°C for 30 s (annealing temperature depending on primers), 72°C for 1 min and a final extension at 72°C for 10 min. PCR products were sequenced using the Macrogen-Europe© (the Netherlands) facility for *Arsenophonus* of Ms, Q from Reunion, *B. afer* and *T. vaporariorum*, and using Genoscreen (Lille, France) for *Arsenophonus* of Q from other locations, ASL and AnSL.

### Phylogenetic analyses

Multiple sequences were aligned using MUSCLE [51] algorithm implemented in CLC DNA Workbench 6.0 (CLC Bio). Phylogenetic analyses were performed using maximum-likelihood (ML) and Bayesian inferences for each locus separately and for the concatenated data set.

JModelTest v.0.1.1 was used to carry out statistical selection of best-fit models of nucleotide substitution [52] using the Akaike Information Criterion (AIC). A corrected version of the AIC (AICc) was used for each data set because the sample size (n) was small relative to the number of parameters (n/K < 40). This approach suggested the following models: HKY for *fbaA*, GTR for *ftsK*, HKY+I for *yaeT* and GTR+I for the concatenated data set. Under the selected models, the parameters were optimized and ML analyses were performed with Phyml v.3.0 [53]. The robustness of nodes was assessed with 100 bootstrap replicates for each data set.

Bayesian analyses were performed as implemented in MrBayes v.3.1.2 [54]. According to the BIC (Bayesian information criterion) estimated with jModelTest, the selected models were the same as for ML inferences. For the concatenated data set, the same models were used for each gene partition. Analyses were initiated from random starting trees. Two separate Markov chain Monte Carlo (MCMC) runs, each composed of four chains, were run for 5 million generations with a “stoprule” option to end the run before the fixed number of generations when the convergence diagnostic falls below 0.01. Thus, the number of generations was 3,000,000 for *FbaA*, 600,000 for *FtsK*, 2, 100,000 for *YaeT* and 1,000,000 for the concatenated data set. A burn-in of 25% of the generations sampled was discarded and posterior probabilities were computed from the remaining trees. Runs of each analysis performed converged with PSRF values at 1.

In addition, *Arsenophonus* strains identified in the present study were used to infer phylogeny on a larger scale with the *Arsenophonus* sequences from various insect species obtained from Duron *et al*. [17]. The GTR+G model was used for both methods (ML and Bayesian inferences) and the number of generations was 360,000 for the Bayesian analysis.

### Recombination analysis

The multiple sequence alignments used in the phylogenetic analysis were also used to identify putative recombinant regions with methods available in the RDP3 computer analysis package [55]. The multiple sequence alignments were analyzed by seven methods: RDP [56], GENECONV [57], Bootscan [58], Maximum Chi Square [59], Chimaera [60], SiScan [61], and 3Seq [62]. The default search parameters for scanning the aligned sequences for recombination were used and the highest acceptable probability (*p* value) was set to 0.001.

### Diversity and genetic analysis

Identical DNA sequences at a given locus for different strains were assigned the same arbitrary allele number (i.e. each allele has a unique identifier). Each unique allelic combination corresponded to a haplotype.

Genetic diversity was assessed using several functions from the DnaSP package [63] by calculating the average number of pairwise nucleotide differences per site among the sequences (π), the total number of mutations (η), the number of polymorphic sites (S) and the haplotype diversity (Hd). The software Arlequin v.3.01 [64] was used to test the putative occurrence of geographical or species structure for the different population groups by an AMOVA (analysis of molecular variance). The analyses partitioning the observed nucleotide diversity were performed between and within sampling sites (countries, localities) or species (*B. tabaci* species, *T. vaporariorum* and *B. afer*). For each analysis, genetic variation was partitioned into the three following levels: between groups (F_CT_), between populations within groups (F_SC_) and within populations (F_ST_). Significance was tested by 10,000 permutations as described by Excoffier *et al*. [64].

## Results

Three bacterial genes *fbaA*, *yaeT* and *ftsK* of *Arsenophonus* were sequenced for 152 Aleyrodidae individuals sampled from different geographical locations and host plants (Figure 1, Table 1). The obtained sequences exhibited a high degree of identity to sequences from the bacterial genus *Arsenophonus* available in the NCBI database (http://www.ncbi.nlm.nih.gov), ranging from 91 to 100% for *fbaA*, 94 to 98% for *yaeT*, and 91 to 100% for *ftsK.* The G-C content varied from 39 to 46% (Table 3), the expected range for these bacteria [65].

**Table 3:** Genetic diversity of ***Arsenophonus fbaA, ftsK*** and ***yaeT*** and concatenated sequences calculated for each group and all individuals.

		*fbaA* (l=366 bp)						*ftsK* (l=251 bp)						*yaeT* (l=289)						3 genes concatenated (l=906)				
		
Group	N	Mean GC%	S	η	π	h	Hd	Mean GC%	S	η	π	h	Hd	Mean GC%	S	η	π	h	Hd	S	η	π	h	Hd
**Ms**	62	39.3	2	2	0.0002	2	0.032	43.4	0	0	0	1	0	38.8	3	3	0.0003	3	0.064	5	5	0.0002	4	0.095
***T. vaporariorum* / Ms**	23	39.3	1	1	0.0002	2	0.087	45.0	0	0	0	1	0	38.8	0	0	0	1	0	1	1	0.0001	2	0.087
**ASL / AnSL**	10	41.6	1	1	0.0015	2	0.533	46.1	20	21	0.018	3	0.6	38.9	8	8	0.0055	2	0.2	29	29	0.0068	4	0.711
**ASL**	10	39.3	0	0	0	1	0	45.0	19	19	0.015	2	0.2	38.7	1	1	0.0007	2	0.2	21	22	0.0051	4	0.711
**Q3**	20	41.8	0	0	0	1	0	45.8	0	0	0	1	0	38.8	2	2	0.0007	2	0.1	2	2	0.0002	2	0.1
**Q2**	26	39.3	0	0	0	1	0	45.2	1	1	0.0011	2	0.271	38.1	0	0	0	1	0	1	1	0.0003	2	0.271

All individuals*	152	39.8	42	45	0.033	9	0.747	44.6	29	30	0.038	9	0.770	38.7	33	35	0.02945	11	0.773	104	110	0.0333	19	0.793

Shown are: mean GC%, number of polymorphic sites including gaps (S), the total number of mutations (**η**),average number of pairwise nucleotide differences per site among the sequences (π), number of haplotypes (h) and haplotype diversity (Hd).

• The total number of individuals includes the singleton *B. afer*.

### Prevalence and co-occurrence of *Arsenophonus*

*Arsenophonus* revealed highly variable prevalences among and within genetic groups and locations (Table 1). Within the Q3 and ASL groups found only in Africa, more than 80% of the individuals were infected with *Arsenophonus*, whereas the prevalence was lower in the AnSL group (50% on average). The infection level was much more variable in Q2 (from 33 to 100%) and Ms (from 4 to 100%). Furthermore, all individuals tested from *T. vaporariorum* (30) and *B. afer* (2) were infected with *Arsenophonus*. Since the sampling was not performed on the same host plants, or in the same locations or countries for a given group, we could not test for the influence of host plant or locality. Based on the three sequenced genes, we could not detect individual co-infection by two lineages of *Arsenophonus* in the same whitefly.

### Allelic variation

Nine alleles were found for both *ftsK* and *fbaA*, and 11 for *yaeT* (Table 4). In these three genes, only 12.1% of the sites showed variation (110/906; Table 3). The observed allelic diversity was not randomly distributed. In fact, strong and significant differentiation (*F*ct = 0.69*, explaining 69% of the total variation in the sample, Table S1 in Additional file 1) was observed between groups of alleles, with each group being mostly associated to a genetic group within the *B. tabaci* complex or the other Aleyrodidae species tested (*T. vaporariorum* or *B. afer*).

**Table 4:** Haplotype distribution among the three sequenced genes of *Arsenophonus* (*fbaA, ftsK, yaeT*).

Haplotype (*B. tabaci* genetic group)	Profile			Number	Frequency (%)
			
	*fbaA*	*ftsK*	*yaeT*		
DATO11(Ms)	6	8	11	59	38.82
BLAPE1 (Q2)	1	5	9	22	14.47
B4-16 (Q3)	4	4	5	19	12.50
co_p1_2 (Tv/Ms)	5	7	10	22	14.47
B1-34 (ASL)	1	2	1	5	3.29
B2-32 (ASL/AnSL)	3	3	2	5	3.29
BLAPE11 (Q2)	1	6	9	4	2.63
B1-21 (ASL)	1	1	1	3	1.97
B1-45 (ASL/AnSL)	2	3	2	3	1.97
B2-37 (ASL)	1	2	4	1	0.66
B1-42 (ASL)	1	3	1	1	0.66
B1-47 (ASL/AnSL)	2	2	2	1	0.66
BE8-23 (ASL/AnSL)	3	3	8	1	0.66
O2-22 (Q3)	4	4	2	1	0.66
PiHarF55 (Ms)	6	8	12	1	0.66
SE616 (Ms)	6	8	14	1	0.66
DIAU8 (Ms)	7	8	11	1	0.66
SaaubF53	8	9	13	1	0.66
Tanza_4.1 (Tv/Ms)	9	7	10	1	0.66

**n haplotypes**	9	9	11	152	100

Number of individuals per haplotype and frequencies are indicated. The name of each haplotype is the name of one of its representatives. The genetic groups of *B. tabaci* associated with the haplotype are indicated in parentheses.

For the *ftsK* locus, we observed indels of two types: a 2-bp insertion found exclusively in the *Arsenophonus* hosted by the Q2 genetic group and a 1-bp deletion found in some ASL and Q2 individuals. These two indels resulted in hypothetical truncated *ftsK* proteins potentially encoding 866 or 884 amino acids, respectively (predicted *ftsK* has 1030 amino acids in *Arsenophonus nasoniae* [Genbank: CBA73190.1]; (Table S2 in Additional file 1).

Among the 152 individuals used in this study, a total of 19 haplotypes of *Arsenophonus* were identified, which is low compared to the theoretical 891 allelic combinations (9 x 9 x 11, 9 alleles for both *ftsK* and *fbaA*, and 11 for *yaeT;* Table 4).

### Recombination analysis

**Figure 2:**
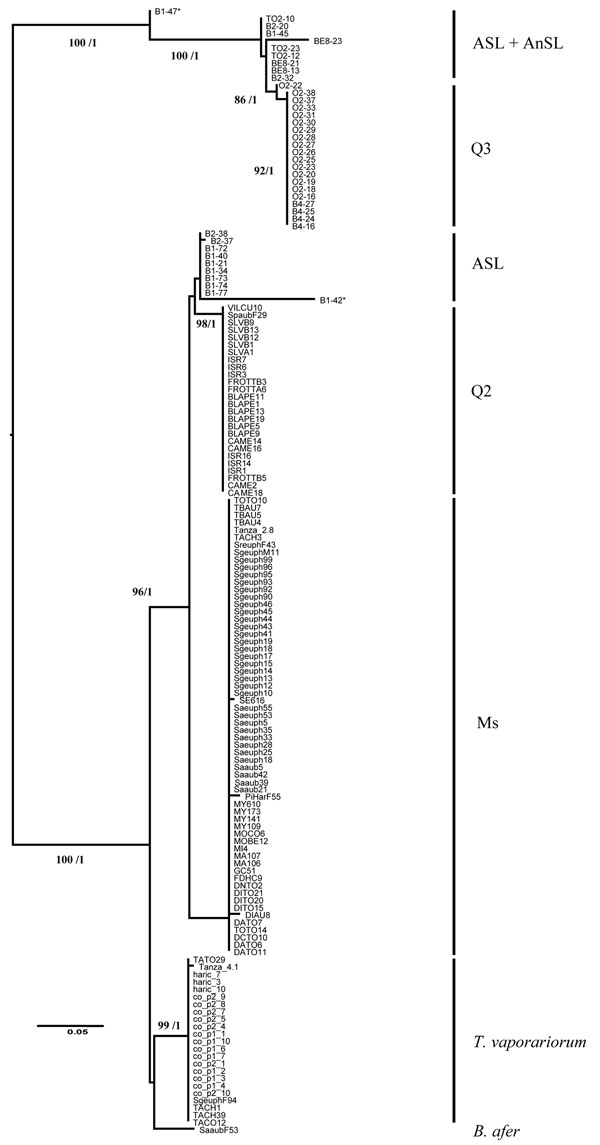
***Arsenophonus* phylogeny constructed using maximum-likelihood (ML) analyses based on the concatenated sequences of three genes: *fbaA*, *ftsK and yaeT*.** The GTR+I evolution model was used to reconstruct this phylogeny. Bootstrap values are shown at the nodes for ML analysis. For nodes also supported by Bayesian inferences, the corresponding posterior probability is shown after the bootstrap value obtained by ML estimations. The tree was midpoint rooted. Recombinant individuals are indicated with an asterisk.

Using the RDP3 package, recombination events were tested for each gene separately and for the concatenated data set using all sequences studied (see Figure 2). No recombination events were detected for any of the gene portions analyzed separately, suggesting that there is no intragene recombination. For the concatenated data set sequences, among the seven algorithms tested, four (GENECONV, Bootscan, Maximum Chi Square, and Chimaera) showed two significant recombination events (Table S3 in Additional file 1). Recombination events were detected in individuals B1-47 and B1-42 (ASL genetic group) for the whole region of the *ftsK* gene (positions 366 to 617 in the concatenated alignment).

Parental-like sequences determined for the recombinant B1-42 were VILCU10 (Q2 genetic group, major parent) and B1-45 (ASL genetic group, minor parent), and parental-like sequences for the recombinant B1-47 were O2-22 (Q3 genetic group, major parent) and B1-34 (ASL genetic group, minor parent). These two recombinant sequences suggest a recombination event between *Arsenophonus* sequence-like of the Q2 and ASL genetic groups for B1-42 and between Q3 and ASL genetic groups for B1-47.

### Phylogenetic inference of relationships

**Figure 3:**
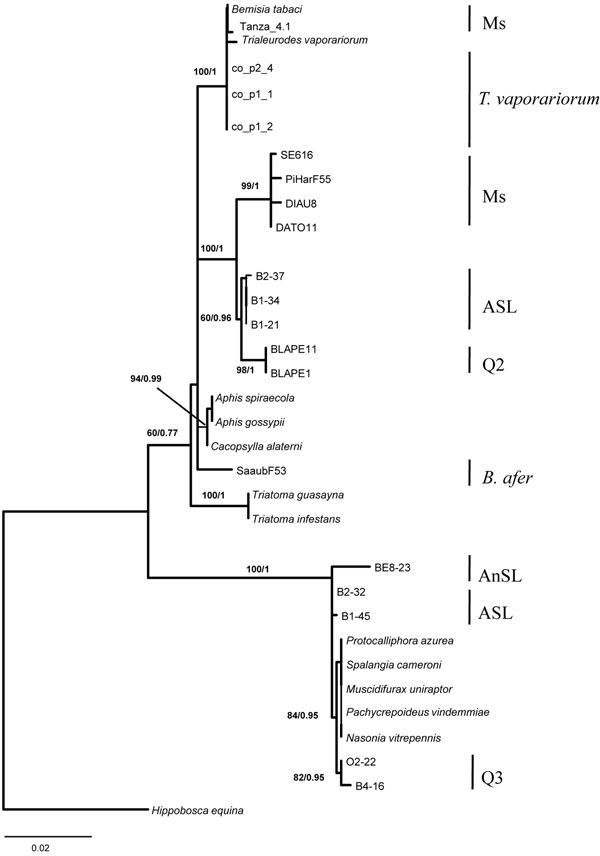
**Global *Arsenophonus* phylogeny constructed with representative haplotype sequences of this study and with *Arsenophonus* sequences from the literature**[17]**[Genbank: GU226783–GU226823].** This tree was constructed using maximum-likelihood (ML) analyses based on the concatenated sequences of the three genes: *fbaA, ftsK* and *yaeT*. The GTR+G evolution model was used to reconstruct this phylogeny, and recombinants were discarded from the analysis (Figure 2). Bootstrap values are shown at the nodes. For nodes also supported by Bayesian inferences, the corresponding posterior probability is shown after the bootstrap value obtained by ML estimations. *Arsenophonus* from *Hippobosca equina* was used as the outgroup. Strains retrieved from the literature are named by their host species and are in italics.

All tree topologies (each gene separately and the combined analysis) were the same with both ML and Bayesian analyses, and we therefore present trees with both bootstrap statistics and Bayesian posterior probabilities (Figures 2, 3; Figure S2 in Additional file 1).

#### Phylogenetic analysis among *Arsenophonus* from *Aleyrodidae*

The phylogenetic trees obtained for each of the three loci were congruent except for the two recombinants (B1-42 and B1-47). Thus, we conducted analyses using the 907-bp concatenated *fbaA*, *ftsK* and *yaeT* sequences.

The concatenated tree (Figure 3) revealed the existence of two highly supported clades composed of six groups and one singleton (the *Arsenophonus* found in *B. afer*, genetically distant from *B. tabaci*; Figure S1 in Additional file 1).

The first clade was composed of Q2, Ms, *Trialeurodes* and some ASL individuals. The second clade was composed of Q3, ASL and AnSL individuals. Interestingly, ASL individuals sampled from the same location and host plant (Burkina Faso, Bobo/Kuinima, Tomato, Marrow; Table 1) were found in both *Arsenophonus* clades, and included the recombinants as well.

The six phylogenetic groups of *Arsenophonus* highly correlated with the *B. tabaci* genetic groups defined on the basis of the mitochondrial *COI*, and with the two other Aleyrodidae species. Indeed, four groups were composed exclusively of individuals belonging to the same genetic group, respectively Ms, ASL, Q3 and Q2. The two other groups included either two distinct *COI* groups of *B. tabaci* ASL and AnSL or individuals from two different host species : *B. tabaci* (with Ms genetic group individuals from Madagascar, Tanzania and Reunion) and *T. vaporariorum* (Tables 3, 4).

Comparative analysis of the genetic divergence of these groups at the three loci (Tables 3, 4) revealed that the group composed of ASL and AnSL individuals is the most polymorphic (π = 0.0068), while the Q2 group is highly homogeneous despite several sampling origins (Table 1). Overall, DNA polymorphism was rather low with an average value of group π means of 0.002.

#### Phylogenetic relatedness of *Arsenophonus* strains from other insects species

The *Arsenophonus* isolates observed in our *B. tabaci* samples proved to be phylogenetically very close to the *Arsenophonus* strains found in other insect species (Figure 3). One clade, composed of *T. vaporariorum*, *B. afer*, the *B. tabaci* groups Ms, Q2, and some individuals belonging to ASL, fell into the *Aphis* sp. and *Triatoma* sp. *Arsenophonus* clade described by Duron *et al*. [17]. The other clade was comprised mainly *Arsenophonus* infecting Hymenoptera (*Nasonia vitripennis*, *Pachycrepoideus vindimmiae*, *Muscidifurax uniraptor*) and the dipteran *Protocalliphora azurea*.

## Discussion

In this paper we report on a survey of the *Arsenophonus* bacterial symbiont in whitefly species, and in particular in *B. tabaci.* The data revealed considerable within-genus diversity at this fine host taxonomic level*.* Previous studies conducted in several arthropod species have found *Arsenophonus* to be one of the richest and most widespread symbiotic bacteria in arthropods [9,15]. However, those studies were performed with *16S rRNA*, which is present in multiple copies in the genome of the bacterium [25] and has proven to be a marker that is highly sensitive to methodological artifacts, leading to an overestimation of the diversity [15].

The phylogenetic analyses performed on concatenated sequences of three *Arsenophonus* genes from whiteflies identified two well-resolved clades corresponding to the two clades obtained in the MLST study performed by Duron *et al*. on a larger insect species scale [17]. One clade was composed of *Arsenophonus* lineages from three *B. tabaci* genetic groups (Ms, ASL, Q2), *T. vaporariorum* and *B. afer*, and strains found in other Hemiptera. The other clade, initially clustering *Arsenophonus* strains found in Hymenoptera and Diptera, also contained whitefly symbionts of the AnSL, ASL and Q3 genetic groups of the *B. tabaci* species complex. This clade thus combines insect hosts from phylogenetically distant taxa. The lineages of *Arsenophonus* from this clade were most likely acquired by whiteflies more recently through lateral transfers from other insect species. The genetic groups of *B. tabaci* represented in this clade all originated from Africa (AnSL, ASL and Q3), which could be explained by horizontal transmission events among groups of *B. tabaci* after a first interspecific transfer of *Arsenophonus* from another insect genus*.* There have been many reports of interspecific horizontal transfers of facultative symbiotic bacteria, suggesting that this phenomenon is frequent in arthropods and probably represents the most common process in the establishment of new symbioses [8]. For example, extensive horizontal transmissions of the reproductive manipulator *Wolbachia* have occurred between insect species [66]. However, horizontal transfers of *Arsenophonus* were poorly documented at the time. Nevertheless, a bacterium called *Candidatus Phlomobacter fragariae*, which is pathogen of strawberry plants, is phylogenetically close to *Arsenophonus* associated with some hemiptera (from cixiids) and more distantly related to psyllid and delphacid secondary endosymbionts [20,67], showing probable evidence of horizontal transfer between plants and insects. Recently Duron *et al*. [17] demonstrated, by phylogenetic analysis and experimental studies, the existence of such horizontal transmission of *Arsenophonus* strains among different wasp species through multi-parasitism. Here we provide indirect phylogenetic evidence of horizontal transmission of *Arsenophonus* among distantly related species that do not have clear intimate ecological contact (via predation or parasitism for instance) and thus have less opportunities for horizontal transfers. This could be explained by the particular features of *Arsenophonus*, most notably its broad spectrum of host species (many insect taxa but also plants) and its ability to grow outside the host [68].

On a lower taxonomic scale, within the whitefly species, 19 haplotypes were identified among the 152 concatenated sequences of *Arsenophonus* obtained in this study. They formed six phylogenetic groups and one singleton corresponding to the *Arsenophonus* strain found in the host species *B. afer*. These groups did not cluster individuals according to host plant or sampling site, and four of them were congruent to the *B. tabaci* genetic groups.

Among the two other phylogenetic groups, one clustered *B. tabaci* individuals that belonged to two strongly diverse genetic groups, ASL and AnSL, which are considered two different species [29] and which were not collected on either the same host plant or in the same country (Burkina Faso and Benin/Togo, respectively). Only some of the ASL individuals belonged to this group, while the others clustered together. These two groups split into the two clades found in whiteflies, which may reflect two separate acquisition events.

The other group of *Arsenophonus* comprised individuals of two whitefly species, *T. vaporariorum* and *B. tabaci* (Ms individuals originated from different countries: Madagascar, Tanzania or Reunion). The *Arsenophonus* strains found in Ms individuals clustered into two groups, but they fell into the same clade (close to Hemiptera). The haplotype diversity of this group was very low, suggesting a recent transfer between *T. vaporariorum* and Ms. One hypothesis is that the exchange of *Arsenophonus* lineages between these two species occurred through their parasitoids, as previously described for *Wolbachia* in planthoppers [69], since *T. vaporariorum* and *B. tabaci* share some parasitoid species (such as *Encarsia* or *Eretmocerus*) and are usually found in sympatry. A second pathway of infection could be through their feeding habit via the plant, as both species are found in sympatry in the field and share the same host plant range. Such a method of symbiont acquisition has been hypothesized for *Rickettsia* in *B. tabaci *[70].

Within the *B. tabaci* species complex, we found, for the first time for *Arsenophonus*, intergenic recombination events in two individuals belonging to the ASL genetic group. The parental-like sequences came from Q2, Q3 and ASL individuals. Although unexpected for intracellular bacteria, homologous recombination has been described in some endosymbiotic bacteria [26,27]. For example, *Wolbachia* showed extensive recombination within and across lineages resulting in chimeric genomes [27]; Darby *et al*. [25] also found evidence of genetic transfer from *Wolbachia* symbionts, and phage exchange with other gammaproteobacterial symbionts, suggesting that *Arsenophonus* is not a strict clonal bacterium, in agreement with the present study. These recombination events may have important implications for the bacteria, notably in terms of phenotypic effects and capacity of adaptation to new hosts, and thus for the bacterial-host association [8], and might prevent the debilitating effects of obligate intracellularity (e.g., Muller’s rachet [71]). In the *Wolbachia* genome, intergenic and intragenic recombinations occur; we detected only intergenic recombination events between *ftsK* and the two other genes in *Arsenophonus*. Surprisingly, we detected indels inducing STOP codons in this gene. These indels, found in all individuals of the Q2 genetic group sampled in Israel, France, Spain, and Reunion, disables the end of the *ftsK* portion sequenced in this study. In bacteria, *ftsK* is part of an operon of 10 genes necessary for cell division [72]. However, a recent study has demonstrated that, in *Escherichia coli*, overexpression of one of the 10 genes of this operon (*ftsN*) is able to rescue cells in which *ftsK* has been deleted [73]. This gene, *ftsN*, is also present in the *Arsenophonus* genome [Genbank: CBA75818.1]. These data suggest that *ftsK* may be not suitable for a MLST approach and other conserved genes should be targeted instead. Future studies should focus on obtaining extensive data related to the specificity of *Arsenophonus*-Q2 interactions. It would be interesting to sample more Q2 individuals infected with *Arsenophonus* to determine the prevalence of this STOP codon in natural populations and its consequences for the bacteria.

## Conclusions

In this study, we found that the diversity of *Arsenophonus* strains in *B. tabaci* corresponds with the diversity observed on a larger scale in insect species. It would be interesting, in further studies, to extend the sampling to more host species in order to get an accurate idea of the diversity of *Arsenophonus* lineages. However, a complete understanding of the *Arsenophonus* phylogeny would require more molecular markers. This could be achieved through the use of other housekeeping genes for the MLST approach or insertion sequences and mobile elements, which is now possible since the genome of *Arsenophonus* has been completely sequenced. We found intergenic recombinations using only three genes, suggesting that such events could be frequent in the *Arsenophonus* genome. Understanding the *Arsenophonus* genomic features is crucial for further research on the evolution and infection dynamics of these bacteria, and on their role on the host phenotype and adaptation. According to these effects on host physiology and phenotype, they could then be potentially exploited in efforts to manipulate pest species such as *B. tabaci*.

## Authors' contributions

All authors made substantial contributions to conception, design, acquisition of data, or analysis and interpretation of data. They were involved in drafting the manuscript and revising it, and have given final approval of the version to be published.

## Competing interests

The authors declare that they have no competing interests.

## Supplementary Material

Additional file 1 **Figure S1. Partial mitochondrial *COI* gene phylogeny of Aleyrodidae individuals used in this study.** The tree was constructed using a Bayesian analysis. Node supports were evaluated by posterior probabilities using the Trn+I+G model. The sequences used in this study are recorded in GenBank as: AnSL Benin (Be8-23) [JF743056], Ms Madagascar (TACH3) [JF743052], Reunion (SPaubF29) [JF743055], Seychelles (SE616) [JF743053] and *Bemisia afer* (Saaub53) [JF743054]. **Figure S2. *Arsenophonus* phylogeny using maximum-likelihood (ML) and Bayesian analyses based on sequences of the three genes *fbaA* (A), *ftsK* (B) and *yaeT* (C).** Different evolution models were used to reconstruct the phylogeny for each gene [*fbaA* (HKY), *ftsK* (GTR), *yaeT* (HKY+I)]. Bootstrap values are shown at the nodes for ML analysis and the second number represents the Bayesian posterior probabilities. **Table S1. Analysis of molecular variance computed by the method of Excoffier *et al. ***[69]** on samples of *Arsenophonus* from several Aleyrodidae species.** Group denomination was according to their hosts, i.e. *Bemisia tabaci:* ASL, AnSL, Q2, Q3, Ms, *Bemisia afer*, *Trialeurodes vaporariorum.* Each species (group) was separated into populations corresponding to location of sampling*.* *p < 0.05. **Table S2. Haplotypes of the three sequenced genes *fbaA* (A), *ftsK* (B), *yaeT* (C) recovered across all 152 samples of Aleyrodidae collected in this study.** Only polymorphic positions are shown, and these are numbered with reference to the consensus sequence. Dots represent identity with respect to reference. The frequency indicates the number of times the haplotype was found in the total sample. *non-synonymous mutations. • Deletion of an A in position 14 for haplotypes B1-21 and BLAPE11 induced a stop codon in position 42 for the analyzed *ftsK* sequence. • Insertion of TC in positions 63-64 for haplotype BLAPE1 & 11 induced a stop codon in position 95 for the analyzed *ftsK* sequence. **Table S3. Recombination in *Arsenophonus*. **Details of the *Arsenophonus* recombination events detected in this study, including parental-like sequences, and p-values for various recombination-detection tests, using RDP3 [60]. Click here for file
