# Supplementary material for: Evidence of diversity and recombination in Arsenophonus symbionts of the Bemisia tabaci species complex
Source: BMC Microbiol. 2012 Jan 18;12(Suppl 1):S10. doi: 10.1186/1471-2180-12-S1-S10 (PMC3287507; doi:10.1186/1471-2180-12-S1-S10)
Supplement: Additional file 1 — Figure S1. Partial mitochondrial COI gene phylogeny of Aleyrodidae individuals used in this study. The tree was constructed using a Bayesian analysis. Node supports were evaluated by posterior probabilities using the Trn+I+G model. The sequences used in this study are recorded in GenBank as: AnSL Benin (Be8-23) [JF743056], Ms Madagascar (TACH3) [JF743052], Reunion (SPaubF29) [JF743055], Seychelles (SE616) [JF743053] and Bemisia afer (Saaub53) [JF743054]. Figure S2. Arsenophonus phylogeny using maximum-likelihood (ML) and Bayesian analyses based on sequences of the three genes fbaA (A), ftsK (B) and yaeT (C). Different evolution models were used to reconstruct the phylogeny for each gene [fbaA (HKY), ftsK (GTR), yaeT (HKY+I)]. Bootstrap values are shown at the nodes for ML analysis and the second number represents the Bayesian posterior probabilities. Table S1. Analysis of molecular variance computed by the method of Excoffier et al. [69] on samples of Arsenophonus from several Aleyrodidae species. Group denomination was according to their hosts, i.e. Bemisia tabaci: ASL, AnSL, Q2, Q3, Ms, Bemisia afer, Trialeurodes vaporariorum. Each species (group) was separated into populations corresponding to location of sampling. *p < 0.05. Table S2. Haplotypes of the three sequenced genes fbaA (A), ftsK (B), yaeT (C) recovered across all 152 samples of Aleyrodidae collected in this study. Only polymorphic positions are shown, and these are numbered with reference to the consensus sequence. Dots represent identity with respect to reference. The frequency indicates the number of times the haplotype was found in the total sample. *non-synonymous mutations. • Deletion of an A in position 14 for haplotypes B1-21 and BLAPE11 induced a stop codon in position 42 for the analyzed ftsK sequence. • Insertion of TC in positions 63-64 for haplotype BLAPE1 & 11 induced a stop codon in position 95 for the analyzed ftsK sequence. Table S3. Recombination in Arsenophonus. Details of the Arsenop [file 1471-2180-12-S1-S10-S1.pdf]

Additional files

Additional file Figure S1 - Partial mitochondrial *COI* gene phylogeny of Aleyrodidae individuals used in this study.

The tree was constructed using a Bayesian analysis. Node supports were evaluated by posterior probabilities using the Trn+I+G model. The sequences used in this study are recorded in GenBank as: AnSL Benin (Be8-23) [JF743056], Ms Madagascar (TACH3) [JF743052], Reunion (SPaubF29) [JF743055], Seychelles (SE616) [JF743053] and *Bemisia afer* (Saaub53) [JF743054].

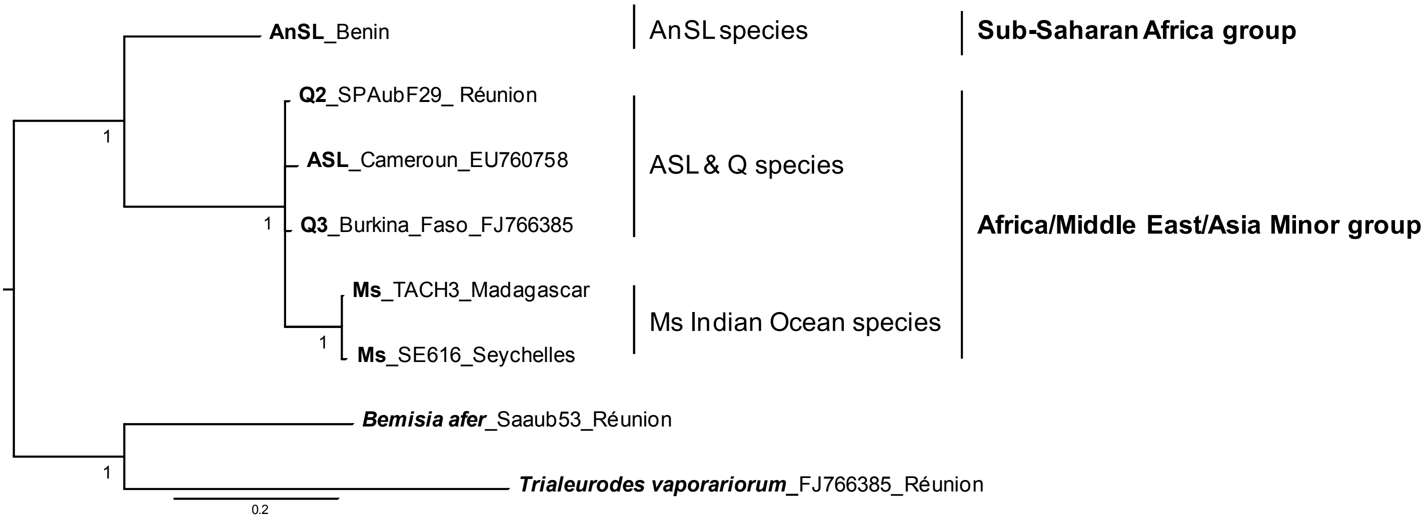

Additional file Figure S2 - *Arsenophonus* phylogeny using maximum-likelihood (ML) and Bayesian analyses based on sequences of the three genes *fbaA* (A), *ftsK* (B) and *yaeT* (C). Different evolution models were used to reconstruct the phylogeny for each gene [*fbaA* (HKY), *ftsK* (GTR), *yaeT* (HKY+I)]. Bootstrap values are shown at the nodes for ML analysis and the second number represents the Bayesian posterior probabilities.

28 A.

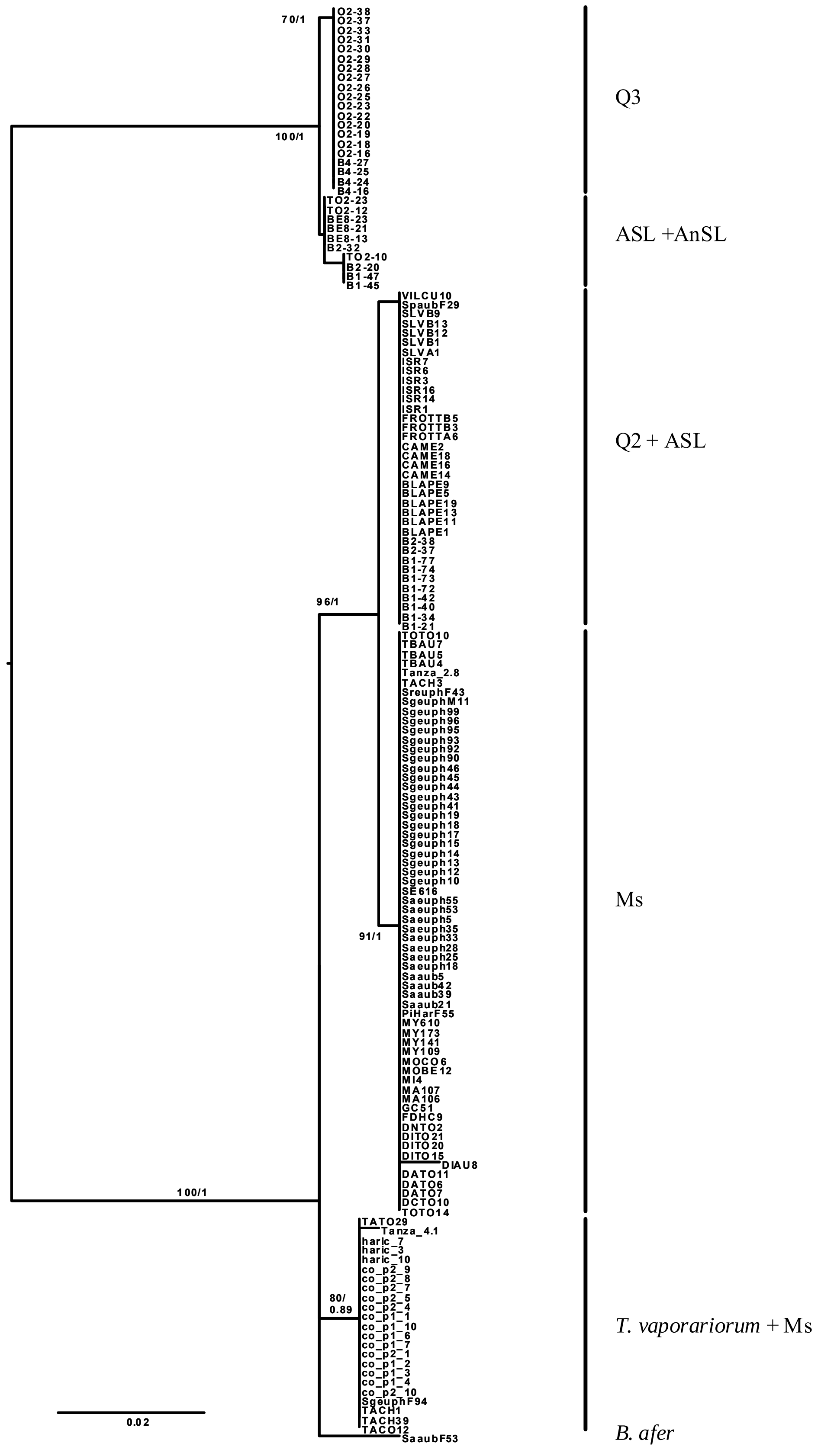

70/1
O2-38
O2-37
O2-33
O2-31
O2-30
O2-29
O2-28
O2-27
O2-26
O2-25
O2-23
O2-22
O2-20
O2-19
O2-18
O2-16
B4-27
B4-25
B4-24
B4-16
100/1
TO2-23
TO2-12
BE8-23
BE8-21
BE8-13
B2-32
TO2-10
B2-20
B1-47
B1-45
VILCU10
SpaubF29
SLVB9
SLVB13
SLVB12
SLVB1
SLVA1
ISR7
ISR6
ISR3
ISR16
ISR14
ISR1
FROTTB5
FROTTB3
FROTTA6
CAME2
CAME18
CAME16
CAME14
BLAPE9
BLAPE5
BLAPE19
BLAPE13
BLAPE11
BLAPE1
B2-38
B2-37
B1-77
B1-74
B1-73
B1-72
B1-42
B1-40
B1-34
B1-21
96/1
TOTO10
TBAU7
TBAU5
TBAU4
Tanza_2.8
TACH3
SreuphF43
SgeuphM11
Sgeuph99
Sgeuph96
Sgeuph95
Sgeuph93
Sgeuph92
Sgeuph90
Sgeuph46
Sgeuph45
Sgeuph44
Sgeuph43
Sgeuph41
Sgeuph19
Sgeuph18
Sgeuph17
Sgeuph15
Sgeuph14
Sgeuph13
Sgeuph12
Sgeuph10
SE616
Saeuph55
Saeuph53
Saeuph5
Saeuph35
Saeuph33
Saeuph28
Saeuph25
Saeuph18
Saaub5
Saaub42
Saaub39
Saaub21
PiHarF55
MY610
MY173
MY141
MY109
MOCO6
MOBE12
MI4
MA107
MA106
GC51
FDHC9
DNTO2
DITO21
DITO20
DITO15
DIAU8
DATO11
DATO6
DATO7
DCTO10
TOTO14
91/1
100/1
TATO29
Tanza_4.1
haric_7
haric_3
haric_10
co_p2_9
co_p2_8
co_p2_7
co_p2_5
co_p2_4
co_p1_1
co_p1_10
co_p1_6
co_p1_7
co_p2_1
co_p1_2
co_p1_3
co_p1_4
co_p2_10
SgeuphF94
TACH1
TACH39
TACO12
SaaubF53
80/
0.89
0.02
Q3
ASL +AnSL
Q2 + ASL
Ms
T. vaporariorum + Ms
B. afer

B.

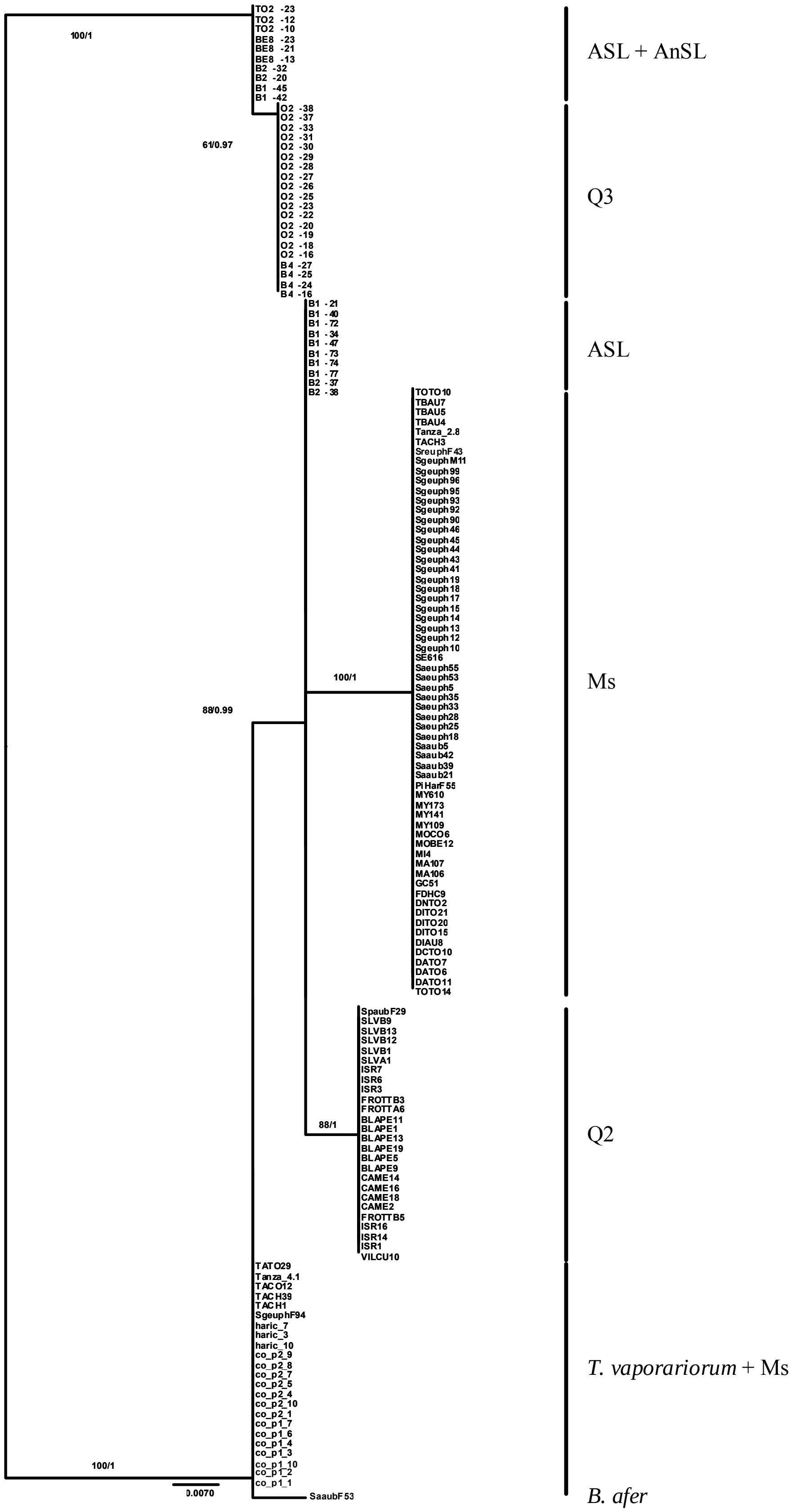
100/1
TO2 -23
TO2 -12
TO2 -10
BE8 -23
BE8 -21
BE8 -13
B2 -32
B2 -20
B1 -45
B1 -42
61/0.97
O2 -38
O2 -37
O2 -33
O2 -31
O2 -30
O2 -29
O2 -28
O2 -27
O2 -26
O2 -25
O2 -23
O2 -22
O2 -20
O2 -19
O2 -18
O2 -16
B4 -27
B4 -25
B4 -24
B4 -16
B1 -21
B1 -40
B1 -72
B1 -34
B1 -47
B1 -73
B1 -74
B1 -77
B2 -37
B2 -38
TOTO10
TBAU7
TBAU5
TBAU4
Tanza_2.8
TACH3
SreuphF43
SgeuphM11
Sgeuph99
Sgeuph96
Sgeuph95
Sgeuph93
Sgeuph92
Sgeuph90
Sgeuph46
Sgeuph45
Sgeuph44
Sgeuph43
Sgeuph41
Sgeuph19
Sgeuph18
Sgeuph17
Sgeuph15
Sgeuph14
Sgeuph13
Sgeuph12
Sgeuph10
SE616
Saeuph55
Saeuph53
Saeuph5
Saeuph35
Saeuph33
Saeuph28
Saeuph25
Saeuph18
Saaub5
Saaub42
Saaub39
Saaub21
PiHarF55
MY610
MY173
MY141
MY109
MOCO6
MOBE12
MI4
MA107
MA106
GC51
FDHC9
DNTO2
DITO21
DITO20
DITO15
DIAU8
DCTO10
DATO7
DATO6
DATO11
TOTO14
100/1
88/0.99
SpaubF29
SLVB9
SLVB13
SLVB12
SLVB1
SLVA1
ISR7
ISR6
ISR3
FROTTB3
FROTTA6
BLAPE11
BLAPE1
BLAPE13
BLAPE19
BLAPE5
BLAPE9
CAME14
CAME16
CAME18
CAME2
FROTTB5
ISR16
ISR14
ISR1
VILCU10
88/1
TATO29
Tanza_4.1
TACO12
TACH39
TACH1
SgeuphF94
haric_7
haric_3
haric_10
co_p2_9
co_p2_8
co_p2_7
co_p2_5
co_p2_4
co_p2_10
co_p2_1
co_p1_7
co_p1_6
co_p1_4
co_p1_3
co_p1_10
co_p1_2
co_p1_1
100/1
0.0070
SaaubF53
ASL + AnSL
Q3
ASL
Ms
Q2
T. vaporariorum + Ms
B. afer

C.

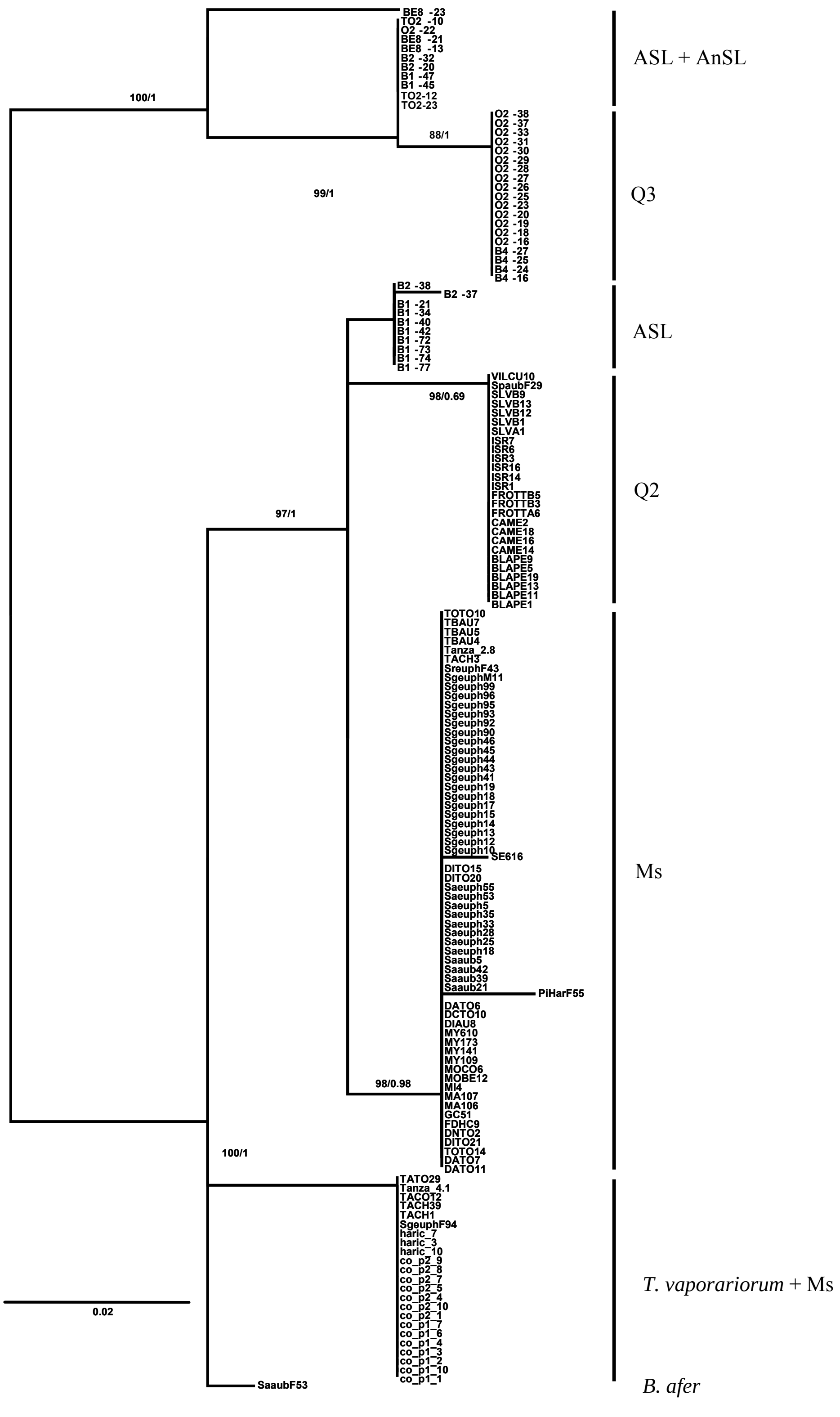
BE8 -23
TO2 -10
O2 -22
BE8 -21
BE8 -13
B2 -32
B2 -20
B1 -47
B1 -45
TO2-12
TO2-23
100/1
O2 -38
O2 -37
O2 -33
O2 -31
O2 -30
O2 -29
O2 -28
O2 -27
O2 -26
O2 -25
O2 -23
O2 -20
O2 -19
O2 -18
O2 -16
B4 -27
B4 -25
B4 -24
B4 -16
88/1
99/1
B2 -38
B2 -37
B1 -21
B1 -34
B1 -40
B1 -42
B1 -72
B1 -73
B1 -74
B1 -77
VILCU10
SpaubF29
SLVB9
SLVB13
SLVB12
SLVB1
SLVA1
ISR7
ISR6
ISR3
ISR16
ISR14
ISR1
FROTTB5
FROTTB3
FROTTA6
CAME2
CAME18
CAME16
CAME14
BLAPE9
BLAPE5
BLAPE19
BLAPE13
BLAPE11
BLAPE1
98/0.69
97/1
TOTO10
TBAU7
TBAU5
TBAU4
Tanza_2.8
TACH3
SreuphF43
SgeuphM11
Sgeuph99
Sgeuph96
Sgeuph95
Sgeuph93
Sgeuph92
Sgeuph90
Sgeuph46
Sgeuph45
Sgeuph44
Sgeuph43
Sgeuph41
Sgeuph19
Sgeuph18
Sgeuph17
Sgeuph15
Sgeuph14
Sgeuph13
Sgeuph12
Sgeuph10
SE616
DITO15
DITO20
Saeuph55
Saeuph53
Saeuph5
Saeuph35
Saeuph33
Saeuph28
Saeuph25
Saeuph18
Saaub5
Saaub42
Saaub39
Saaub21
PiHarF55
DATO6
DCTO10
DIAU8
MY610
MY173
MY141
MY109
MOCO6
MOBE12
MI4
MA107
MA106
GC51
FDHC9
DNTO2
DITO21
TOTO14
DATO7
DATO11
98/0.98
100/1
TATO29
Tanza_4.1
TACOT2
TACH39
TACH1
SgeuphF94
haric_7
haric_3
haric_10
co_p2_9
co_p2_8
co_p2_7
co_p2_5
co_p2_4
co_p2_10
co_p2_1
co_p1_7
co_p1_6
co_p1_4
co_p1_3
co_p1_2
co_p1_10
co_p1_1
SaaubF53
0.02
ASL + AnSL
Q3
ASL
Q2
Ms
T. vaporariorum + Ms
B. afer

Additional file Table S1 - Analysis of molecular variance computed by the method of Excoffier *et al.* [69] on samples of *Arsenophonus* from several Aleyrodidae species.

Group denomination was according to their hosts, i.e. *Bemisia tabaci:* ASL, AnSL, Q2, Q3, Ms, *Bemisia afer, Trialeurodes vaporariorum.* Each species (group) was separated into populations corresponding to location of sampling. **p* < 0.05.

Source of variation	d.f.	Sum of squares	Percentage of variation	F stat
**Between groups**	6	38.86	69.01	FCT : 0.69*
**Between populations (countries/sampling sites) within groups**	11	2.31	2.11	FSC : 0.07
**Within populations**	134	18.70	28.88	FST : 0.71*
**Total**	151	59.87		

Additional file Table S2 - Haplotypes of the three sequenced genes *fbaA* (A), *ftsK* (B), *yaeT* (C) recovered across all 152 samples of Aleyrodidae collected in this study.

Only polymorphic positions are shown, and these are numbered with reference to the consensus sequence. Dots represent identity with respect to reference. The frequency indicates the number of times the haplotype was found in the total sample. *non-synonymous mutations.

A. *fbaA*. Haplotypes	Position in sequence Frequency	6	12	15	20	24	27	44	51	54	57	66	81	83	84	99	107	110	111	120	131	156	157	180	210	228	237	240	243	255	261	267	268	291	297	303	306
Consensus		T	G	A	C	C	G	C	G	A	G	G	G	C	T	G	T	A	A	T	A	A	T	T	T	G	A	G	T	T	T	G	C	A	T	G	A
B1-21	36	.	.	.	.	.	.	.	.	.	.	.	T*	.	.	.	.	.	.	.	.	.	C	.	.	.	.	.	.	.	.	A*	A	.	.	.	.
B1-45	4	C	A	C	.	T	.	.	A	G	.	T	.	.	G	.	.	C*	T	C	.	G	.	C	G	A	C	A	A	C	C	.	.	C	G	C	C
B2-32	6	C	A	C	.	T	.	.	A	G	.	T	.	.	G	.	.	C*	T	C	.	G	.	C	G	A	C	A	A	C	C	.	.	T	G	C	C
B4-16	20	C	A	C	.	T	.	.	A	G	.	T	.	.	G	.	.	C*	T	C	.	G	.	C	G	A	C	A	A	C	C	.	.	T	G	C	C
co_p1_1	22	.	.	.	.	.	T*	.	.	.	.	.	.	.	.	A	.	.	.	.	.	.	.	.	.	.	.	.	.	.	.	.	.	.	.	.	.
DATO11	61	.	.	.	.	.	.	T*	.	.	.	.	.	.	.	.	.	.	.	.	.	.	C	.	.	.	.	.	.	.	.	A*	A	.	.	.	.
DIAU8	1	.	.	.	.	.	.	T*	.	.	.	.	.	T*	.	.	A*	.	.	.	.	.	C	.	.	.	.	.	.	.	.	A*	A	.	.	.	.
Tanza_4.1	1	.	.	.	.	.	T*	.	.	.	.	.	.	.	.	A	.	.	.	.	G*	.	.	.	.	.	.	.	.	.	.	.	.	.	.	.	.
SaaubF53	1	.	.	.	A*	.	.	.	.	.	A	A	.	.	.	.	.	.	.	.	.	.	.	.	.	.	.	.	.	.	.	.	.	.	.	.	.

fbaA. Haplotypes	Position in sequence Frequency	321	328	342	345	362	363
Consensus		G	G	C	C	A	A
B1-21	36	.	.	.	.	.	.
B1-45	4	A	.	T	T	T*	G
B2-32	6	A	.	T	T	T*	G
B4-16	20	A	.	T	T	C*	G
co_p1_1	22	.	.	.	.	.	.
DATO11	61	.	.	.	.	.	.
DIAU8	1	.	.	.	.	.	.
Tanza_4.1	1	.	.	.	.	.	.
SaaubF53	1	.	A*	.	.	.	.

B. ***ftsK.*** **Haplotypes**	**Position in sequence Frequency**	**1**	**6**	**13**	**14**	**16**	**22**	**40**	**52**	**55**	**63**	**64**	**66**	**67**	**75**	**84**	**101**	**104**	**108**	**111**	**121**	**129**	**186**	**195**	**196**	**198**	**201**	**204**	**211**	**219**	**237**	**247**
Consensus		T	C	A	A	G	G	C	G	T	–	–	T	C	G	A	C	G	C	C	G	T	A	C	C	T	C	G	T	G	T	A
B1-21	3	.	.	.	–•	.	.	.	.	.	–	–	.	.	.	.	.	.	.	.	.	.	.	.	.	.	.	.	.	.	.	.
B1-34	7	.	.	.	.	.	.	.	.	.	–	–	.	.	.	.	.	.	.	.	.	.	.	.	.	.	.	.	.	.	.	.
B1-42	10	C	.	G	C	.	C	T	T	C	–	–	.	.	A	G	.	.	.	T	.	C	C	T	.	C	T	T	C*	A	C	C*
B4-16	20	C	.	G	C	A	C	T	T	C	–	–	.	.	A	G	.	.	.	T	.	C	C	T	.	C	T	T	C*	A	C	C*
BLAPE1	22	.	G*	.	.	.	.	.	.	.	C	T■	.	.	.	.	.	C	.	.	.	.	.	.	.	.	.	.	.	.	.	.
BLAPE11	4	.	G*	.	–•	.	.	.	.	.	C	T■	.	.	.	.	.	C	.	.	.	.	.	.	.	.	.	.	.	.	.	.
co_p1_1	23	.	.	.	.	.	.	T*	.	.	–	–	.	.	.	.	.	.	.	.	.	.	.	.	.	.	.	.	C*	.	.	.
DATO11	62	.	.	.	.	.	.	.	.	.	–	–	.	.	.	.	T*	.	T	.	A*	.	.	.	T*	.	.	.	.	.	.	.
SaaubF53	1	.	.	.	.	.	.	T*	.	.	–	–	G	A	.	.	.	.	.	.	.	.	.	.	.	.	.	.	C*	.	.	.

C. ***yaeT.*** **Haplotypes**	**Position in sequence Frequency**	**6**	**10**	**12**	**23**	**26**	**28**	**44**	**57**	**66**	**91**	**101**	**114**	**137**	**140**	**156**	**160**	**164**	**176**	**191**	**197**	**204**	**225**	**230**	**236**	**243**	**245**	**254**	**257**	**258**	**269**	**272**	**275**	**278**
Consensus		G	C	C	G	T	T	G	G	C	C	C	G	T	A	C	C	T	T	T	G	A	C	G	C	C	T	A	C	C	A	A	G	G
B1-21	9	.	.	.	.	.	.	.	.	.	.	.	.	.	.	.	.	.	.	.	.	.	.	.	.	.	.	G	.	.	.	.	A	.
B1-45	10	.	.	.	.	G	.	.	.	.	T*	.	.	A	T	.	.	C	C	.	A	G*	.	A	A	.	C	.	T	.	T	G	.	.
B2-37	1	.	.	.	.	.	.	.	.	.	.	.	.	.	.	.	A*	.	.	.	.	.	.	.	.	.	.	.	.	.	.	.	A	.
B4-16	19	.	.	.	.	G	.	.	.	.	T*	G*	.	A	T	.	.	C	C	.	A	G*	.	A	A	.	C	.	T	T*	T	G	.	.
BE8-23	1	.	.	.	.	G	.	.	.	T*	T*	.	A*	A	T	.	.	.	.	.	A	G*	.	A	.	.	.	.	T	.	T	.	.	A
BLAPE1	26	.	.	T	A*	.	.	.	A*	.	.	.	.	.	.	.	.	.	.	.	.	.	.	.	.	.	.	G	.	.	.	.	.	.
Co_p1_1	23	.	T*	.	.	.	.	.	.	.	.	.	.	.	.	.	.	.	.	C	.	T*	A*	.	.	.	C	.	.	.	.	.	.	.
DATO11	60	A*	.	.	.	.	.	.	.	.	.	.	.	.	.	G*	.	.	.	.	.	.	.	.	.	.	.	G	.	.	.	.	.	.
PiHarF55	1	A*	.	.	.	.	C*	T	.	.	.	.	.	.	.	G*	.	.	.	.	.	.	.	.	.	.	.	G	.	.	.	.	.	.
SaaubF53	1	.	.	.	.	.	.	.	.	A*	.	.	.	.	.	.	.	.	.	.	.	G*	.	.	.	.	C	.	.	.	.	.	.	.
SE616]	1	A*	.	.	.	.	.	.	.	.	.	.	.	.	.	G*	.	.	.	.	.	.	.	.	.	T*	.	G	.	.	.	.	.	.

• Deletion of an A in position 14 for haplotypes B1-21 and BLAPE11 induced a stop codon in position 42 for the analyzed *ftsK* sequence.

▪ Insertion of CT in position 63-64 for haplotypes BLAPE1 & 11 induced a stop codon in position 95 for the analysed *ftsK* sequence.

Additional file Table S3 - Recombination in *Arsenophonus*.

Details of the *Arsenophonus* recombination events detected in this study, including parental-like sequences, and p-values for various recombination-detection tests, using RDP3 [60].

				Detection Methods				
	Recombinant Sequence(s)	Minor Parental Sequence(s)	Major Parental Sequence(s)	RDP	GENECONV	Bootscan	Maxchi	Chimaera
Recombination events in Figure 3	B1-42	B1-45	VILCU10	1,00E-05	7,40E-04	1,00E-05	6,00E-05	3,30E-05
	B1-47	B1-34	O2-22	3,50E-05	1,00E-03	2,00E-05	1,10E-03	5,80E-04
